# Supplementary material for: Learning curve and surgical outcome of robotic assisted colorectal surgery with ERAS program
Source: Sci Rep. 2022 Nov 29;12:20566. doi: 10.1038/s41598-022-24665-w (PMC9709162; doi:10.1038/s41598-022-24665-w)
Supplement: Supplementary file 1 — Supplementary Information 1. [file 41598_2022_24665_MOESM1_ESM.zip › LSC Raw data-Ñ[▒K/098/N_ñw╝╨░O▒Kñσ.pdf]

RCC / RCC

臺中榮民總醫院
手術室護理記錄

[redacted]

基本資料

- 室別： 二醫手術室-31室，手術科別： CRS，手術類別： 常規，日期： 2020/10/21
- 進入時間： 1455 ； 麻醉開始時間： 1455 ； Time Out時間： 1555 ； 劃刀時間： 1556 ； 手術結束時間： 1930 送出時間： 1950 ；
- 麻醉方式： GE

  診斷： Malignant tumor of rectum

  手術名稱： 1.Low anterior resection with colo-anal anastomosis2.Ileostomy3.Laparoscopy4.

  手術局部用藥： nil

  術前預防性抗生素：
  - 抗生素名稱一： Veterin 1GM IVD由麻醉護理師給予
  - 時間： 1550
  - 上次簽章: 張仟品

  ，追加預防性抗生素：
- 手術醫師： 蔣鋒帆，林昌霖，陳映菁，楊捷儒，李研永
- 麻醉醫師： VS 施涵薰
-
  - 刷手護士A ： 吳雅華，時間： 1455-1500，接替刷手一： 陳昱穎，時間： 1500--1800，接替刷手二： 王毓均，時間： 1800
  - 流動護士A ： 張仟品，時間： 1455-1730，接替流動一： 陳思帆，時間： 1730-

皮膚狀況

A-V Shunt： 無

術前： 不完整

部位(術前)：

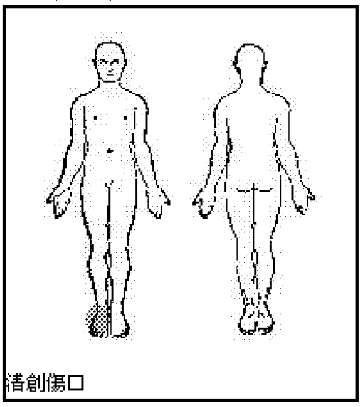

記錄者： 上次簽章: 張仟品

術後： 不完整

部位(術後)：

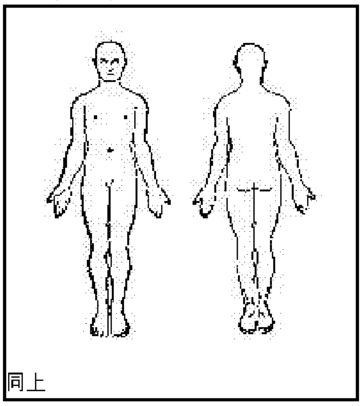

記錄者： 陳思帆

手術準備

手術臥式 單一手術臥式

-
  - 手術臥式： 截石臥式
  - 使用附件： 溫毯 38 ℃，頭圈，腳架
  - 皮膚準備： 10%Aq Betadine，Hibiscrub，2% Chlorhexidine
  - 電燒： 使用電燒: 電極片位置： 其它： 電燒墊，記錄者： 上次簽章: 張仟品

止血帶T： 無

植入物

- 植入物名稱/號碼/數量/部位：surgiwrap100*130mm/壹/colon

, 記錄者： 上次簽章: 陳思帆

標本送檢

- 細菌培養 無
- 細胞學 無
- 冰凍切片 無
- 病理標本
  組織名稱一：rectum
  ,
  組織名稱二：small intestine
  ,
  組織名稱三：IMA root
  , 更多組織名稱
  - 組織名稱四：distal cutend
- 截肢 無

標本交付 標本： 肆 袋，標本： 0 盒，標本： 0 瓶，細胞學： 0 管，Culture Tube： 0 管，被交付醫師： CR 陳映菁，

- 記錄者： 陳思帆
- 時間： 2000

傷口術後敷料

傷口敷料：

- 請於圖上標示部位：

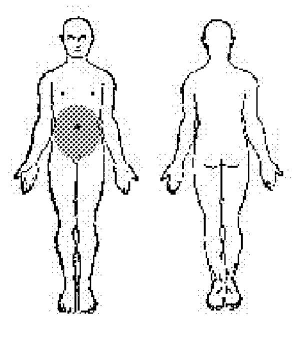

腹部伍切口

- 傷口敷料：mepore覆蓋

導管

導管	天數	導管資訊	插入日期	插入時間	更換日期	拔除日期	拔除時間	拔除原因	預計拔除/更換	預計拔除/更換日期
Foley(1)	1	號碼:16 Fr.; 部位:膀胱;	2020/10/21	1550						
Rod,Stoma Supporting (1)	1	號碼:一個 ; 部位:右腹;	2020/10/21	1840						
A-line(1)	1	部位:左手; 註記:22G;	2020/10/21	1550						

用物計數

- 手術中出血量： minimal c.c.
- 手術中尿量： 250 c.c.
-
  - 其它： 無

用物計數： 多切口同時縫合

- 1.器械： 正確，2.縫針： 正確，3.刀片： 正確，4.紗布： 正確，5.腹部墊： 未使用，6.紗球： 未使用，7.PEANUTS ： 未使用，8.紗條： 未使用，9.絲帶： 正確，10.針頭： 未使用，11.Vessel loop： 未使用，12.Boots： 未使用，13.Jelco： 未使用，14.棉條： 未使用，
  - 第一次：
    - 刷手護士 ： 王毓均
    - 流動護士： 上次簽章: 陳思帆
    - 時間： 1845

  ,
  - 第二次：
    - 刷手護士 ： 王毓均
    - 流動護士： 上次簽章: 陳思帆
    - 時間： 1855

  ,
  - 第三次：
    - 刷手護士 ： 王毓均
    - 流動護士： 上次簽章: 陳思帆
    - 時間： 1905

備註

備註：NIL

- 記錄者： 陳思帆 上次簽章: 陳思帆
- 時間： 2000

填寫護理人員 陳思帆

正式摘要

RCC / RCC

doctorRank:B
